# A virtual screen identified C96 as a novel inhibitor of phosphatidylinositol 3-kinase that displays potent preclinical activity against multiple myeloma *in vitro* and *in vivo*

**DOI:** 10.18632/oncotarget.1657

**Published:** 2014-05-22

**Authors:** Juan Tang, Jingyu Zhu, Yang Yu, Zubin Zhang, Guodong Chen, Xiumin Zhou, Chunhua Qiao, Tingjun Hou, Xinliang Mao

**Affiliations:** ^1^ Department of Pharmacology, College of Pharmaceutical Sciences, Soochow University, Suzhou, China; ^2^ Cyrus Tang Hematology Center, Jiangsu Institute of Hematology, The First Affiliated Hospital, Soochow University, Suzhou, China; ^3^ Department of Medicinal Chemistry, College of Pharmaceutical Sciences, Soochow University, Suzhou, China; ^4^ Department of Oncology, The First Affiliated Hospital of Soochow University, Suzhou, China; ^5^ College of Pharmaceutical Sciences, Zhejiang University, Hangzhou, China; ^6^ Collaborative Innovation Center of Hematology, Suzhou, China

**Keywords:** C96, apoptosis, PI3K/AKT signal pathway, multiple myeloma, virtual screen

## Abstract

The phosphatidylinositol 3-kinase (PI3K)/AKT signaling pathway is emerging as a promising therapeutic target for multiple myeloma (MM). In the present study, we performed a virtual screen against 800,000 of small molecule compounds by targeting PI3Kγ. C96, one of such compounds, inhibited PI3K activated by insulin-like growth factor-1 (IGF-1), but did not suppress IGF-1R activation. The cell-free assay revealed that C96 preferred to inhibit PI3Kα and δ, but was not active against AKT1, 2, 3 or mTOR. C96 inhibited PI3K activation in a time- and concentration-dependent manner. Consistent with its inhibition on PI3K/AKT, C96 downregulated the activation of mTOR, p70S6K, 4E-BP1, but did not suppress other kinases such as ERK and c-Src. Inhibition of the PI3K/AKT signaling pathway by C96 led to MM cell apoptosis which was demonstrated by Annexin V staining and activation of the pro-apoptotic signals. Furthermore, C96 displayed potent anti-myeloma activity in a MM xenograft model in nude mice. Oral administration of 100 mg/kg bodyweight almost fully suppressed tumor growth within 16 days, but without gross toxicity. Importantly, AKT activation was suppressed in tumor tissues from C96-treated mice, which was consistent with delayed tumor growth. Thus, we identified a novel PI3K inhibitor with a great potential for MM therapy.

## INTRODUCTION

Multiple myeloma (MM) is a malignancy of plasma cells, a type of white blood cells normally responsible for producing antibodies. This disease is characterized by impaired hematopoesis, renal dysfunction, bone destruction, and eventual death [1]. MM is the second most common hematological malignancy in the U.S. and constitutes 1% of death related to cancers. Currently, chemotherapy is one of the major treatments for MM, including newly marketed proteasome inhibitors and immunomodulators, however, MM remains incurable, and the median survival period is 3–5 years with conventional treatments [2]. All current drugs inherit limited efficacy and/or severe toxicity or adverse effects [3, 4], therefore, developing novel agents is in an urgent need, especially in targeted drug discovery.

Among the “druggable” targets in MM, the phosphoinositide-3-kinase (PI3K)/AKT signal pathway is of particular interest. PI3K/AKT plays a critical role in regulating a myriad of cell signals involved in cell proliferation, migration and survival of MM [5–7]. There are four isoforms in the Class I PI3K superfamily, including PI3Kα, β, δ and γ, all of which have been demonstrated to be overactivated in MM cells [8, 9]. PI3K dysregulation is closely associated with overexpressed growth factors, such as insulin-like growth factor-1 (IGF-1), and cytokines, such as interleukin-6 (IL-6) [10, 11]. These extracellular signals bind to specific receptors (e.g. IGF-1R and IL-6R), and then activate PI3K, which catalyzes the production of PI(3,4,5)P3, a key second messenger, that further mediates AKT activation and downstream signals in the MM pathophysiology [12]. The PI3K/AKT signal pathway is highly associated with chemoresistance and poor prognosis of MM, thus being developed as a promising target for MM therapy [8].

With the advances of combinatorial chemistry, cancer biology, informatics technology and bioengineering, several important strategies and techniques have been developed, including structural optimization, high throughput screen, and computer-based *in silico* screen [13]. A virtual screen belongs to the *in silico* screens, which utilizes high-performance computing to identify possible drug candidates which are most likely to bind to a drug target, typically a protein receptor or an enzyme. Compared with traditional high throughput screens, virtual screens are reliable, cost-effective and time-saving [14]. In the present study, we performed a virtual screen against 800,000 small molecule compounds from ChemBridge and Specs Chemicals libraries by using PI3Kγ as the subject. PI3Kγ is frequently expressed in MM cells [8, 9], and several inhibitors of PI3Kγ have been developed in the preclinical stages for MM therapy, such as CAL-101, IPI-145, BEZ235, and PI-103 [15], which established a rationale for the discovery of PI3K inhibitors. More importantly, the molecular interaction of small chemical inhibitors and PI3Kγ has been clearly elucidated [16, 17]. Therefore, PI3Kγ is a well established target for the discovery of PI3K inhibitors. After several rounds of screens and cell- and mouse-based studies, C96, one of these compounds, was identified as a promising candidate for MM therapy.

## RESULTS

### C96 inhibits PI3K activity

**Figure 1:**
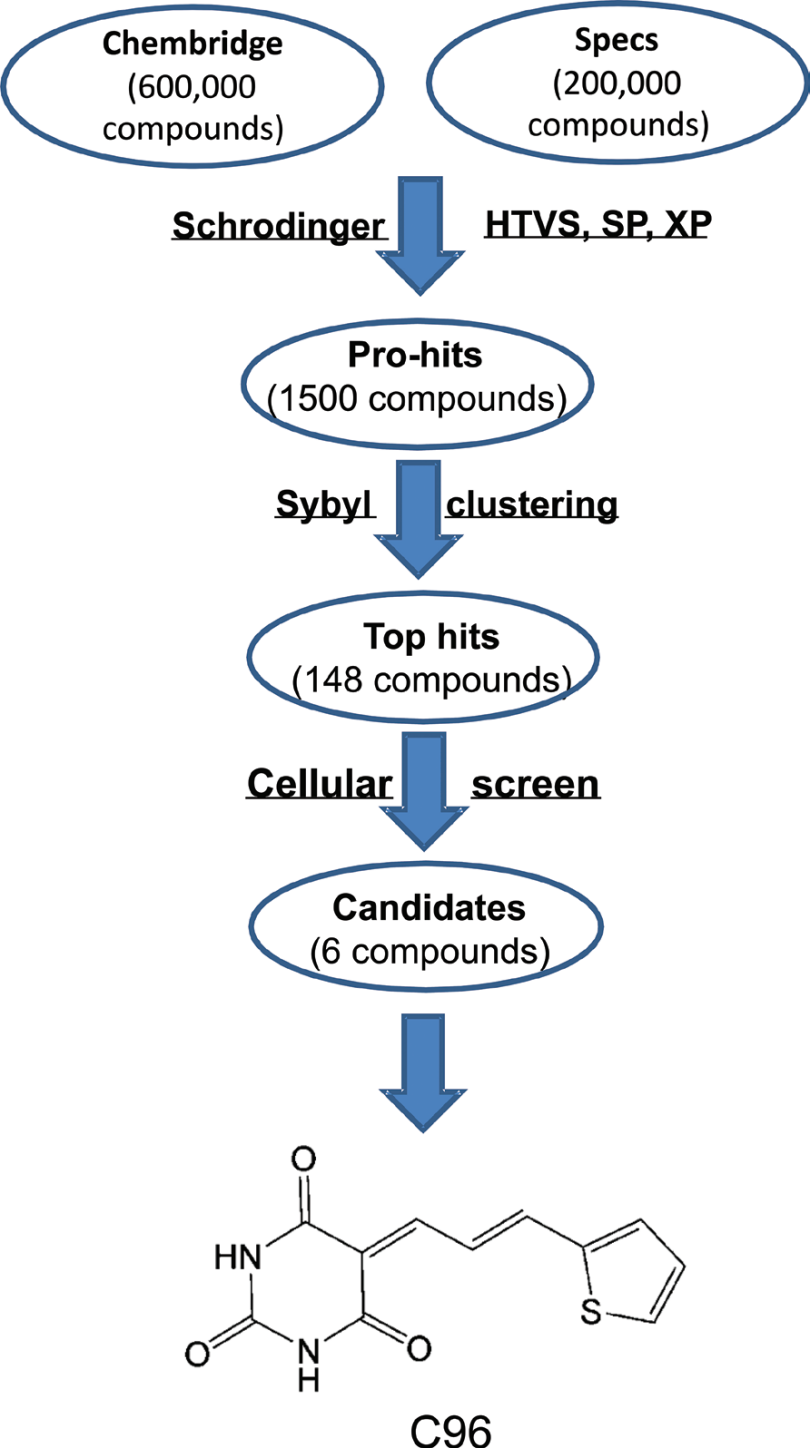
The virtual screening workflow C96 was generated from a virtual screen using PI3Kγ as the subject against 800,000 compounds in total from Specs and ChemBridge Chemicals. The molecular docking and scoring were accomplished by using the Schrodinger (Glide), HTVS, SP, and XP modes, followed by Sybyl clustering. Top hits were then verified at the cell-based experiments and singled out for further studies.

**Figure 2:**
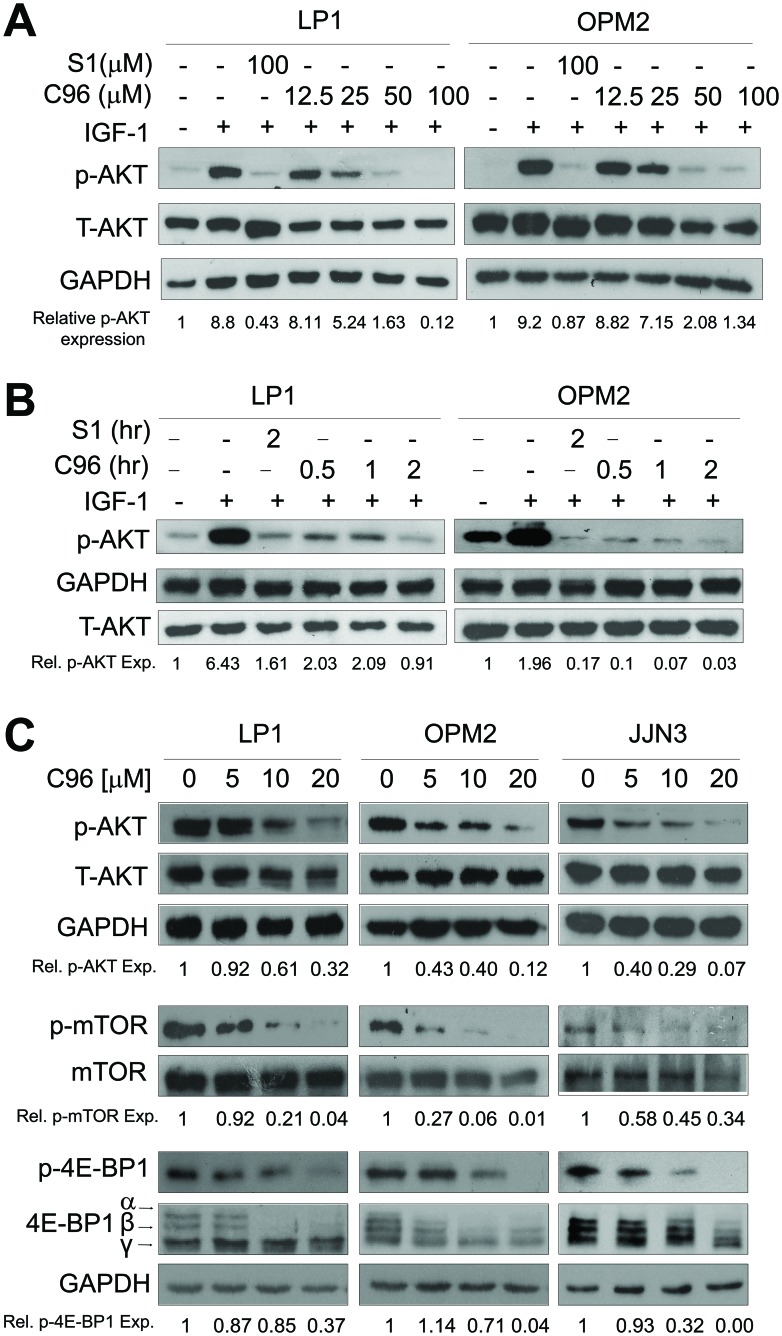
C96 inhibits AKT and mTOR signaling (**A**) LP1 and OPM2 were starved overnight, then treated with C96 at the indicated concentrations, or 100 μM of S14161 for 2 hrs, followed by IGF-1 (100 ng/mL) for 15 min. Cells were collected for the analysis of the expression of p-AKT and total AKT (T-AKT) by immunoblotting. (**B**) LP1 and OPM2 cells were starved overnight, then treated with C96 (50 μM) for different time periods, or S14161 (100 (μM) for 2 hrs, followed by IGF-1 (100 ng/mL) for 15 min. Cells were for the analysis of the expression of p-AKT and T-AKT by immunoblotting. (**C**) LP1, OPM2, and JJN3 cells were treated with C96 at the indicated concentrations for 24 hrs. Cell lysates were prepared and subjected to immunoblotting assay against p-AKT, AKT, p-mTOR, mTOR, p-4E-BP1, and 4E-BP1. GAPDH was used as a loading control.

Because C96 was identified from a virtual screen by using PI3Kγ as the target against 800,000 compounds as shown in Figure 1, we subsequently verified its inhibitory activity against PI3K in MM cells using AKT phosphorylation as a readout. MM cell lines LP1 and OPM2 were starved overnight before being treated with C96 (0–100 μM) or S14161 (100 μM, a positive control [6]) for a short period (2 hrs), followed by IGF-1 stimulation for 15 min. Immunoblotting revealed that C96 significantly suppressed AKT phosphorylation in a concentration-dependent manner in the presence of IGF-1 but had no effects on total AKT expression, which was similar to the effects of S14161, the proven PI3K inhibitor [6] (Figure 2A). In LP1 cells, C96 at 25 μM and 50 μM led to a 50% and 90% decrease in AKT phosphorylation, respectively, in the 2-hr treatment. AKT phosphorylation was also markedly decreased by C96 in OPM2 cells which does not express PTEN, a negative modulator of the PI3K signaling pathway (Figure 2A, right panel). In the time-course study, AKT activation was suppressed by C96 at 50 μM within 0.5 hrs (30 min) (Figure 2B). These studies suggested that C96 inhibited PI3K activity in a time- and concentration-dependent manner.

The PI3K/AKT plays a critical role in regulating a myriad of downstream effectors [18], of which the most prominent ones are mTOR/p70S6K/4E-BP1. Many PI3K inhibitors eventually modulate cell proliferation and survival by disrupting this particular pathway [19]. To examine whether PI3K inhibition led to deregulation of the mTOR signaling pathway, we further measured the changes of mTOR, p70S6K and 4E-BP1 in MM cell lines LP1, OPM2, and JJN3 in the presence of C96. As shown in Figure 2C, C96 downregulated the phosphorylation levels of these proteins in all examined cell lines in a concentration-dependent manner.

### C96 does not inhibit phosphorylation of other kinases

**Figure 3:**
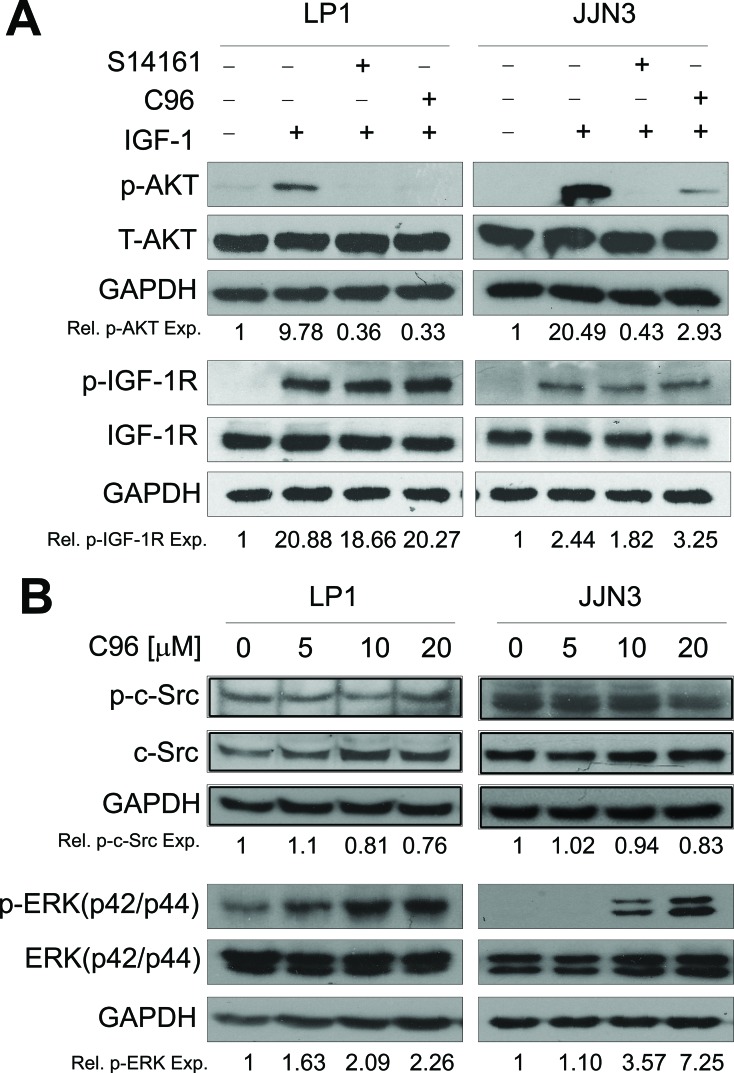
C96 inhibits AKT but not IGF-1R, ERK or c-Src kinase activation (**A**) LP1 and JJN3 cells were starved overnight, then treated with 100 (μM of C96 or S14161 for 2 hrs, followed by IGF-1 (100 ng/mL) for 15 min. After incubation, cells were harvested and the expression of p-AKT, p-IGF-1R, total AKT (T-AKT), and IGF-1R proteins were detected by immunoblotting. (**B**) LP1 and JJN3 were treated with C96 at indicated concentrations for 24 hrs followed by immunoblotting assay for the expression of p-Src, c-Src, p-ERK, and ERK. GAPDH was used as a loading control.

The above studies demonstrated that C96 inhibited the PI3K/AKT signaling pathway, whether it affects activities of other kinases was not known. To elucidate this specificity, we first evaluated its effects on IGF-1R because IGF-1R as a receptor tyrosine kinase activates PI3K in the presence of IGF-1. To exclude the potential effects of C96 on IGF-1 signaling, LP1 and JJN3 cells were starved overnight followed by C96 or S14161 treatment for 2 hrs, and IGF-1 stimulation for 15 min. As expected, IGF-1 triggered AKT phosphorylation but it was suppressed by both C96 and the proven PI3K inhibitor S14161 without affecting total AKT expression (Figure 3A). In this treatment, IGF-1R phosphorylation was sharply induced by IGF-1, but it was not affected by C96 (Figure 3A). This result suggested that C96 inhibited PI3K activity independent of IGF-1R signaling. Next, we evaluated other kinases including non-receptor kinase c-Src and a PI3K parallel kinase ERK which transduces external cellular signals. Both LP1 and JJN3 cells were treated with C96 at increasing concentrations that inhibited AKT and mTOR activation (Figure 2C). However, C96 did not decrease the phosphorylation level of neither c-Src nor ERK (Figure 3B). These results thus suggested that C96 probably mainly inhibited PI3K/AKT/mTOR signaling pathway.

**Figure 4:**
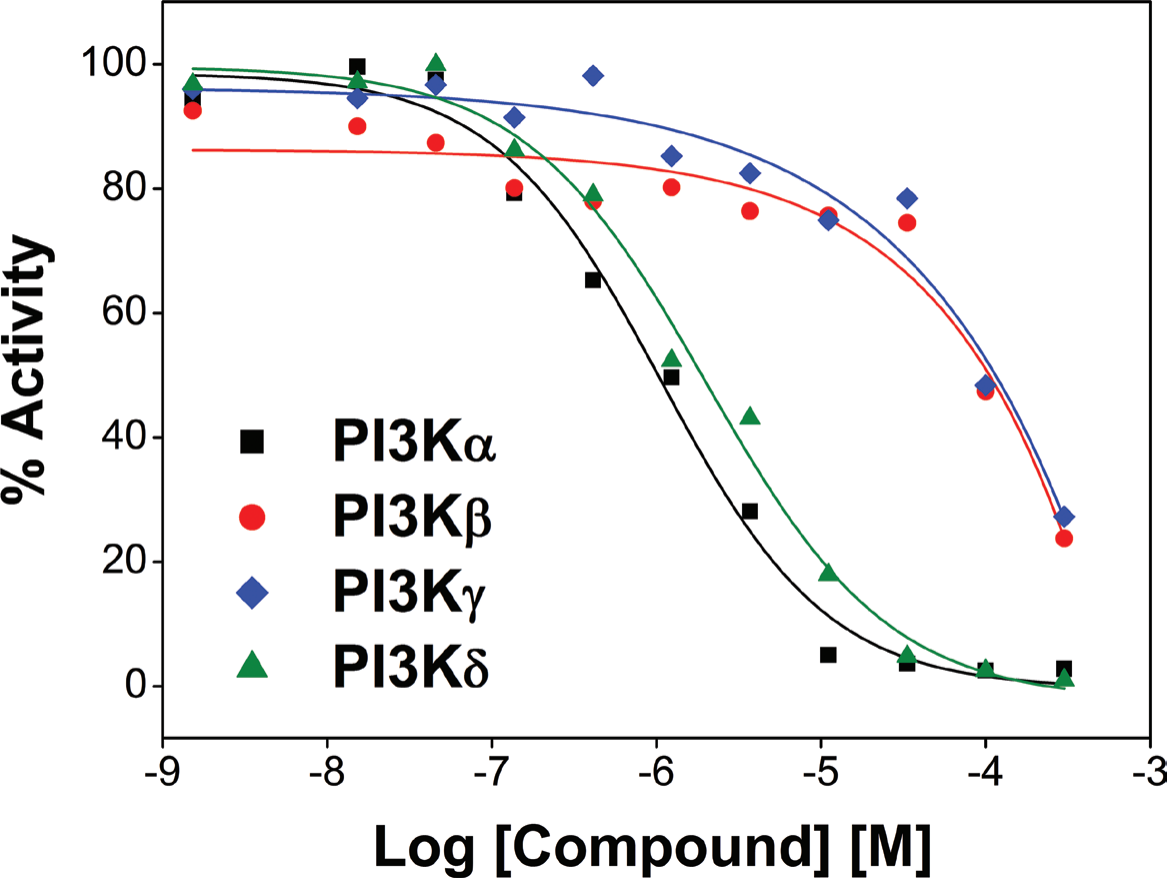
C96 inhibits PI3K activity PI3K activity analyses in an *in vitro* cell-free system. Increasing concentrations of S14161 were incubated with the PI3K isoforms α, β, δ, and γ, respectively. Activity of each kinase was determined with HotSpot technology as described in “Kinase activity in cell-free assay”.

To confirm this hypothesis, we next evaluated the effects of C96 on PI3K family (PI3Kα, β, δ, and γ) and associated enzymes including AKT1, 2, 3 and mTOR, in a cell-free enzymatic system as reported previously [6]. It turned out that C96 preferred to inhibit PI3Kα and δ with an IC_50_=5.41 and 7.05 μM, respectively, and less activity against PI3Kβ and γ (IC_50_=134 and 116 μM, respectively) as shown in Figure 4, but displayed no significant effects on AKT1, 2, 3, or mTOR because all enzymatic activities were higher than 50% even at 300 μM concentration, and their IC_50_ values were not detectable (Supplemental Table 1). Taken together, all the above studies suggested C96 was a preferred inhibitor of PI3K, especially the α and δ isoforms, thus suppressing the PI3K/AKT signaling pathway.

### C96 inhibits proliferation and induces apoptosis of MM cells

**Figure 5:**
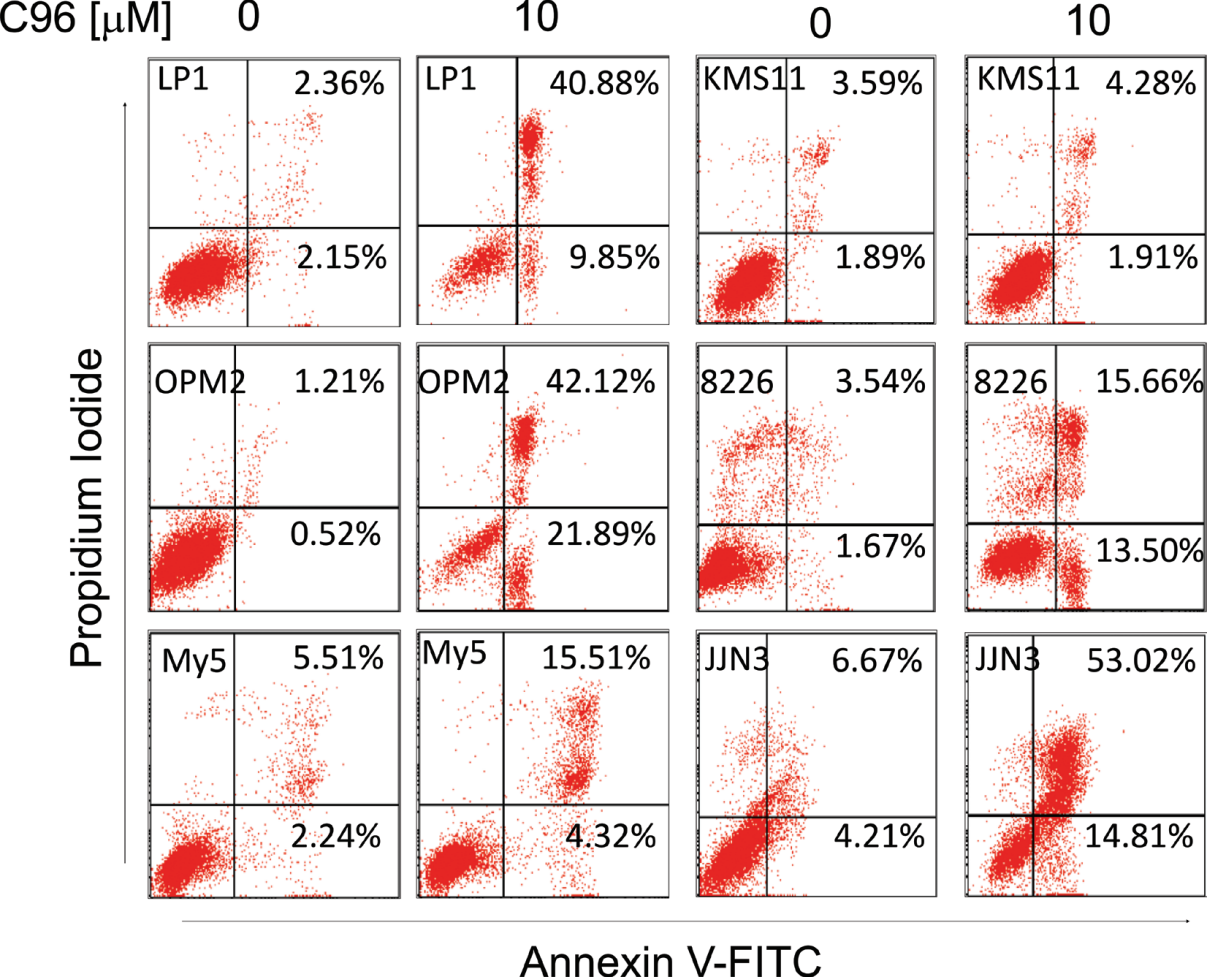
C96 induces MM cell apoptosis MM cell lines LP1, OPM2, OCI-My5 (My5), KMS11, RPMI-8226 (8226), and JJN3 were treated with C96 at 0 or 10 μM for 24 hrs, followed by propidium iodide and Annexin V-FITC staining and flow cytometric analysis.

**Figure 6:**
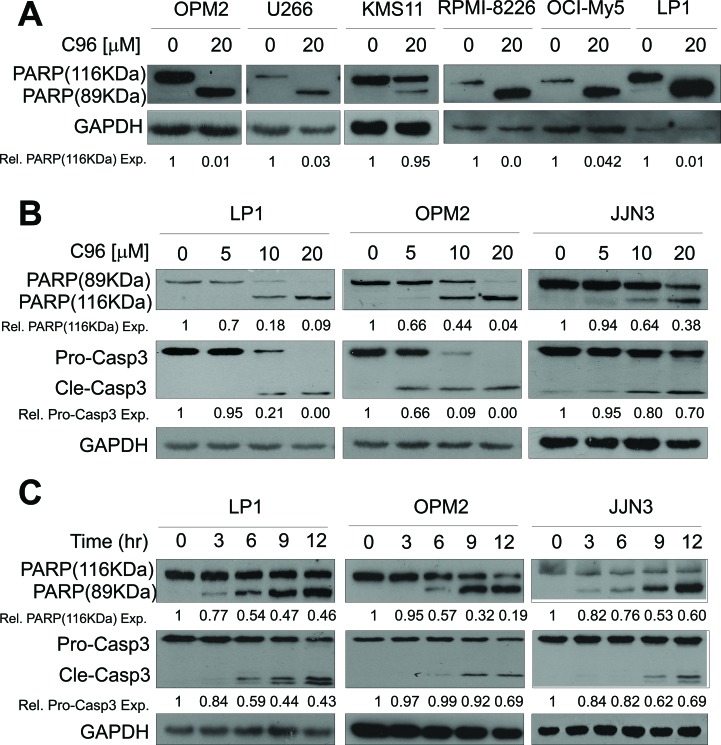
C96 activates apoptotic signaling in MM cells (**A**) OPM2, U266, KMS11, RPMI-8226, OCI-My5, and LP1 cells were treated with C96 (20 μM) for 24 hrs. Cell lysates were then prepared and subjected to immunoblotting assay against apoptosis-associated proteins PARP, and apoptotic executive enzyme pro-caspase-3 (Pro-Casp3). GAPDH was used as a loading control. (**B**) LP1, OPM2, and JJN3 were treated with C96 at the indicated concentrations for 12 hrs, followed by immunoblotting assay for pro-caspase-3 (Pro-Casp3), cleaved caspase-3 (Cle-Casp3), and PARP. GAPDH was used as a loading control. (**C**) LP1, OPM2, and JJN3 were treated with C96 at 10 μM for different time points, followed by immunoblotting assay for caspase-3 and PARP. GAPDH was used as a loading control.

Because PI3K/AKT signaling is critical in modulating MM cell proliferation and survival, while interference with this pathway will lead to cell death and regression of MM tumors [6, 8], we next asked whether C96 could induce MM cell apoptosis and inhibited MM cell proliferation. MTT assays indicated that C96 suppressed cell proliferation and reduced cell viability of a panel of MM cell lines including LP1, OPM2, JJN3, OCI-My5, RPMI-8226, U266, and KMS11 within 72 hrs (Supplemental Figure 1). Next, we measured the induction of apoptosis by C96 in MM cell lines. Apoptotic cells are featured with inside-out expression of phosphatidylserine which is specifically identified by Annexin V, a cellular protein in the annexin group [20]. Therefore, to measure the apoptotic level, C96-treated cells were stained with Annexin V-FITC and propidium iodide followed by flow cytometry analysis. As shown in Figure 5, 20 to 60% of cells underwent apoptosis in the treatment of C96 at 10 μM within 24 hrs. This result was confirmed by the cleavage of PARP, a biomarker of cell apoptosis (Figure 6A). For example, less apoptotic cells were induced by C96 in KMS11 cells in the Annexin V staining assay (Figure 5), the cleavage level of PARP in this cell line was also lower than that seen in other cell lines, such as OPM2 (Figure 6A). We next examined the apoptotic signaling pathway in the treatment with C96 with increasing exposure periods or concentrations. Three cell lines LP1, OPM2, and JJN3 were treated with C96 at 0, 5, 10, or 20 μM for 24 hrs or 10 μM for 3, 6, 9 and 12 hrs. Immunoblotting analysis revealed that C96 induced cleavage of PARP and caspase-3 in a time- and concentration-dependent manner (Figure 6C). All the above results demonstrated that C96 induced MM cell apoptosis.

Next, we evaluated the effects of C96 on MM cell growth by measuring viable cells after starvation/IGF-1 stimulation in the presence of C96. As shown in Supplemental Figure 2A, IGF-1 stimulated MM cell growth, but it was suppressed by C96. We also examined the effects of C96 on MM cell migration upon IGF-1 stimulation. In all three cell lines (LP1, OPM2, and JJN3), LP1 and JJN3 failed to but OPM2 cells could migrate through the 8-micrometer transwell membrane in the presence of IGF-1, and this migration was partly suppressed by C96 (Supplemental Figure 2B). These data suggested that C96 inhibited MM cell mobility in the presence of IGF-1 but it was probably determined by individual cell lines because not all MM cells could migrate through the membrane.

### C96 delays tumor growth in a MM xenograft model in association with the inhibition of the PI3K signaling pathway

**Figure 7:**
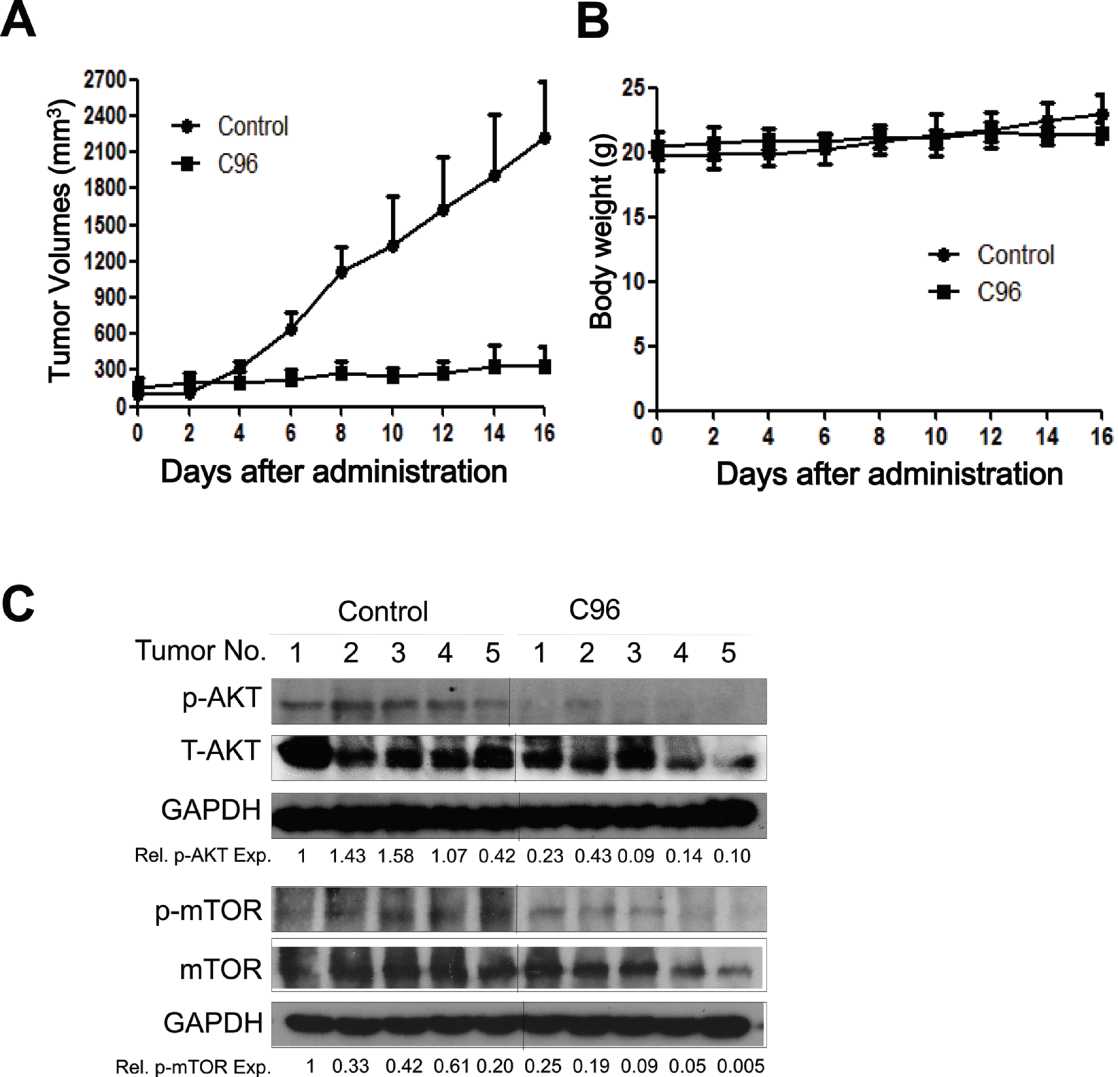
C96 delays myeloma tumor growth in a xenograft mouse model (**A**) JJN3 cells were injected subcutaneously into nude mice with a density of 30 million cells/site/mouse. When tumors were palpable, mice (n=5/group) were orally given C96 (100 mg/kg body weight) in PBS containing 10% Tween 80 and 10% DMSO daily for continuous 16 days. Tumor volumes were monitored every other day. (**B**) Mouse body weight was monitored every other day. (**C**) At the end of the experiment, tumor samples from each group were subjected to immunoblotting analysis for the expression levels of p-AKT, T-AKT, p-mTOR and mTOR with specific antibodies. GAPDH was used as a loading control.

To further evaluate the anti-myeloma efficacy of C96 *in vivo*, a human multiple myeloma xenograft model was established by subcutaneous injection of JJN3 into nude mice. When these tumors were palpable, mice were randomly divided into two groups (n=5) and one of them were orally administrated C96 (100 mg/kg body weight, 1/10 of the oral LD_50_), the other one were given the same volume of vehicle on a daily base for continued 16 days. Tumor sizes and body weight were monitored every other day. As shown in Figure 7A, C96 delayed MM tumor growth during the experimental period. In contrast, the tumors in the vehicle group grew in a steady rate. However, the 16-day treatment of C96 had minimal effects on body weight (Figure 7B), and there were no gross toxicity observed in all mice treated with C96 (data not shown).

We also measured the changes of the phosphorylation status of AKT and mTOR, two key proteins in the PI3K signaling pathway, in the myeloma tumor tissues. Immunoblotting indicated that both AKT and mTOR phosphorylation was decreased in the myeloma tumor tissues excised from C96-treated mice (Figure 7C), which were consistent with MM tumor growth treated by C96. Therefore, C96 delayed tumor growth of myelomas *in vivo*, which was associated with the inhibition of the PI3K signaling pathway and was consistent with the *in vitro* studies.

## DISCUSSION

In the present study, we identified C96 as a potential PI3K inhibitor via virtual screening and subsequent cellular and mice studies against MM.

The PI3K-centered signaling pathway has significance in MM pathophysiology, chemotherapy, as well as clinical prognosis. Consistent with these findings, blockade of the PI3K/AKT signaling pathway with chemical or genetic methods leads to MM cell apoptosis and MM tumor regression [21]. Therefore, targeting PI3K is a key strategy for MM therapy [22]. In the last two decades, robot-assisted large scale targeted drug screens have been significantly implemented due to the advancements in bioinformatics, informative technologies, molecular cancer biology, and synthetic chemistry[13, 23, 24]. In terms of PI3K, the elucidation of crystal structures of PI3K isoforms including co-crystallized ligands and apo-structures deposited in the Protein Data Bank (PDB) has made virtual screening possible [14, 25]. As an attractive possible filter, a virtual screen offers an opportunity to minimize the wet laboratory workload [26]. Using these structure-based virtual screening and extensive docking studies, several PI3K inhibitors have been identified [14, 26, 27]. In the present study, we performed a virtual screen against PI3Kγ because this isoform has been most studied and its crystal structure has been well defined [16, 28]. Using PI3Kγ as the screening subject against the ChemBridge and Specs chemical libraries, we identified C96 as a potential candidate that was further verified in both cellular and mice models. Consistent with the prediction, C96 inhibits PI3K activation and blocks the PI3K/AKT signaling pathway thus inducing MM cell apoptosis. However, the enzymatic assay in a cell-free PI3K system showed that C96 prefers to inhibit PI3Kα and δ although it was screened out against the γ isoform. This is very interesting but it is highly possible. Firstly, all isoforms share a similar structure of the ATP binding pocket, therefore, there are many important pan-Class I PI3K inhibitors [29]. Secondly, the virtual screen is based on the theoretical interactions between compounds and specific enzymes, however, the actual interactions may vary in the cell-free and in the cell-based assays [30]. After all, the real interaction is determined by the specific physical and chemical properties of the compounds and the specific structure of the receptor enzymes [30]. In the case of C96, it is probable that C96 fits better in the α and δ active pockets in the native status, such as in cells or in the purified enzymes. We also believe a preferred inhibitor of the α and δ PI3K isoforms merits further investigation because these two isoforms are overexpressed in MM cells but less expressed in normal blood cells [8]. More importantly, the γ isoform is required in T cell functions and in the migration of leukocytes, in particular neutrophils and macrophages [31]. Therefore, the preference of C96 on the α and δ isoforms will be more important for the potential application for the treatment of MM. This finding also indicated that the virtual screens must be coupled with wet laboratory investigations, especially at the cellular and at the animal levels. After all, the virtual screen is a theoretical prediction, and the cellular and animal studies must be performed because the “real” human being system is very complicated.

In MM cells, the PI3K signaling is particularly important because it is modulated by various extracellular stimulators, such as the growth factor IGF-1, and the cytokine IL-6, both of which are frequently over-expressed and secreted in MM cells thus stimulating PI3K activation [10, 11]. IGF-1R activation leads to sustained activation of PI3K thus increasing cell proliferation and survival [32]. Therefore, PI3K inactivation could be due to the effects on the suppression of these membrane signals, such as IGF-1R and IL-6R activation [33]. Some PI3K signaling transduction can be blocked by inactivating IGF-1R and/or IL-6R. However, C96 displays no inhibitory effect on IGF-1R phosphorylation but markedly inhibits AKT activation, suggesting that C96-mediated PI3K inhibition is not due to its effects on the IGF-1R signaling. More importantly, C96 downregulates PI3K and its subsequent signaling transduction, including the AKT/mTOR pathway, however, its shows no inhibitory effects on other kinases such as c-Src and ERK, suggesting that C96 prefers to inhibit the PI3K/AKT signaling transduction. This is consistent with the cell-free assay in which C96 inhibits the catalytic activity of PI3K but it does not inhibit kinases such as AKT1, 2, 3 or mTOR associated with PI3K. Although C96 has not inhibition on the catalytic activity of AKT or mTOR, it suppressed the phosphorylation of AKT, mTOR, p70S6K, and 4E-BP1, which is probably attributed to PI3K inhibition. Downregulated PI3K/AKT signal transduction leads to suppressed downstream signals, such as the phosphorylation of mTOR and p70S6K, which has been reported previously [5, 34]. In tumor tissues from a mouse xenograft model established with human MM cell line JJN3, both AKT and mTOR activation was also decreased by C96, which is consistent with studies at the the cellular level, therefore, C96-delayed myeloma tumor growth is consistent with its inhibition on PI3K. These results thus collectively concluded that C96 is a preferred inhibitor of PI3K.

In the last decade, a lot of PI3K inhibitors have been developed and some have shown great potencies in preclinical studies against varied cancer types [18, 29], of which some belong to thiazolidinones, 2-aminothiazoles, or 2-morpholinothiazoles. By comparing with those already known PI3K inhibitors, C96 is featured with a novel chemical structure, therefore it probably represents a novel class of PI3K inhibitors. Moreover, C96 displays a very potent activity *in vivo* but without marked toxicity. The EC_50_s against PI3K alpha and delta in the cell-free enzymatic system are around 5 μM, which is acceptable. More importantly, C96 showed great potency in killing MM cells at low micromolar concentrations and suppresses the growth of MM xenograft in nude mice at a dose less than 1/10 LD_50_. At this dose, C96 did not show marked toxicity to mice but suppressed tumor growth more than 90% within 16 days. This is very essential because some known PI3K inhibitors are able to suppress PI3K activity at low nanomolar levels, but they need to reach a concentration up to 10 μM to induce MM cell death. For example, BAY80-6946 displays a high activity against all PI3K isoforms with IC_50_s from 0.5 to 6.4 nM in the cell-free enzymatic system, it requires more than 1000 nM to inhibit MM cell proliferation [9]. Another case is CAL-101, one of the most potent inhibitor of PI3Kγ and δ, whose IC_50_s in inhibiting PI3K activity are around 2.5 nM, however, it needs more than 10 μM to induce MM cell death [8]. These data indicated that cell-free potency is not consistent with its biological activity, and C96 might have better metabolic parameters than some known PI3K inhibitors.

In a summary, by a virtual screen and further studies at both cellular and mice models, C96 was identified as a novel potent PI3K inhibitor, especially against PI3Kα and δ. Because of its efficacy in both *in vitro* and *in vivo* and undetectable toxicity *in vivo*, C96 can be developed as a promising PI3K inhibitor for the treatment of MM, but clinical relevance should be further investigated.

## MATERIALS AND METHODS

### Cell culture

MM cell lines OPM2, RPMI-8226, U266, and KMS11 were obtained from the American Type Culture Collection (Rockville, MD, USA). OCI-My5, LP1, and JJN3 were kindly provided by Dr. Aaron Schimmer from Ontario Cancer Institute, Toronto, Canada. All cell lines were maintained in Iscove's modified Dulbecco's medium (IMDM, Hyclone) supplemented with 10% fetal bovine serum (Invitrogen, CA), penicillin (100 units/mL) and streptomycin (100 μg/mL), and were grown at 37°C in an incubator supplied with 5% CO_2_.

### Chemicals

C96 was purchased from ChemBridge Corporation, San Diego, CA. IGF-1 was purchased from PeproTech [5]. Annexin V-FITC, propidium iodide, MTT or 3-(4,5-dimethylthiazol-2-yl)-2,5-diphenyltetra-sodium bromide were purchased from Sigma (St Louis, MO).

### Virtual screening

The crystal structure of PI3Kγ (PDB entry: 3APC) [35] from the RCSB Brookhaven Protein Data Bank was used as the initial structure for the virtual screening. The databases of 200,000 compounds from Specs (Delft, The Netherlands, http://www.specs.net/) and 600,000 compounds from ChemBridge (San Diego, United States, http://www.chembridge.com) were used as the initial sources for the screen against PI3Kγ [14]. The molecular docking calculations were accomplished by using the Glide module in Schrödinger (version 9.0). According to the binding fitness, each compound was assigned a score. Compounds with the highest scores were moved to the next screen with a stricter condition. All structures were docked and scored using the Glide HTVS, SP, XP modes step by step and 1,500 compounds were returned as pro-hits. Then, chemical similarity clustering of the pro-hits was performed to maximize the chemical diversity of the selected compounds for biological assay using the Selector Module in SYBYL 8.1 and 148 compounds as top-hits were obtained. Lastly, all these 148 compounds were purchased from ChemBridge or Specs, and were applied for cell-based and mouse-based evaluation. C96, 5-(3-(2-thienyl)-2-propen-1-ylidene)-2,4,6(1H,3H,5H)-pyrimidinetrione, was chosen for further investigation. The detailed flow chart for the virtual screening was shown in Figure 1.

### Growth inhibition assay

MM cells were dispensed in 96-well plates at a density of 8×10^3^ cells per well and treated with increasing concentrations of C96 for 72 hrs, and the growth inhibitory effect of C96 on MM cells was assessed by an MTT assay as reported previously [6]. To show the effect of C96 on cell growth in the presence of growth factor IGF-1, MM cells were first maintained in IMDM media containing 0.5% FBS for 12 hrs, followed by C96 treatment alone or in combination with 100 ng/mL of IGF-1. Cells were then incubated for another 24 hrs, relative cell proliferation was analyzed by an MTT assay.

### Flow cytometric analysis of apoptosis

MM cells treaded with C96 at concentrations from 0 to 20 μM, 24 hrs later, cells were stained with Annexin V-FITC and PI according to the manufacturer's instruction (Sigma, St Louis, MO). Apoptotic cells were measured on a flow cytometer (FACSCalibur, Becton Dickinson) as reported as previously [36].

### Kinase activity in cell-free assays

Kinase activity in the presence of C96 was performed by using the HotSpot technology (Reaction Biology Corp., Malvern, PA, USA) [6]. Briefly, kinases (PI3Kα, β, δ, γ, AKT1, 2, 3, and mTOR) and substrates were diluted in reaction buffer [6]. Subsequently, 5 nL of serially diluted C96 (in pure DMSO) was delivered into diluted kinase and substrate mixture by using Echo 550 (LabCyte Inc. Sunnyvale, CA). The reaction was started by adding ^33^P-ATP (hot ATP) into the reaction mixture (the final concentration was 10 μM) and stopped after 2 hrs incubation at room temperature. The un-reacted hot ATP was washed away before detection.

### Immunoblotting analysis

Cells were treated with or without C96 for 24 hrs before being applied to lysate preparation in a lysis buffer (50 mM Tris-HCI, pH 7.4, 1% NP-40, 0.5% Na-deoxycholate, and 0.1% SDS, 150 mM NaCl, 2 mM EDTA, 2 mM Na_3_VO_4_, and 5 mM NaF) [6]. Equal amounts (30 μg) of total proteins were subjected to sodium dodecyl sulfate–polyacrylamide gel electrophoresis (SDS-PAGE) separation, followed by immunoblotting analyses with specific antibodies. Antibodies against PARP, caspase-3, AKT, p-AKT(S473), 4E-BP1, p-4E-BP1(S65), IGF-1R, p-IGF-1R, ERK(p42/p44), and p-ERK(p42/p44) were purchased from Cell Signaling Technologies. Antibodies against mTOR, p-mTOR(S2448), p70S6K, p-p70S6K(S424), and GAPDH were obtained from Abgent (Suzhou, China). Horseradish peroxidase-conjugate secondary antibodies against mouse or rabbit and GAPDH were purchased from Abgent. All immunoblotting signals were further analyzed with Quality One Quality One software (Bio-Rad, Berkeley, CA, USA).

### Cell migration analysis by the transwell assay

This assay was performed as described previously [37]. Briefly, MM cells OPM2, JJN3 and LP1 were serum starved overnight and then re-suspended in 70 μL of IMDM medium/0.5% fetal calf serum with or without C96. These cells were then loaded onto polycarbonate membranes (8-μm pore size) separating 2 chambers of a transwell (Corning Costar, Cambridge, MA). Medium/0.1% FCS (500 μL) containing agonist IGF-1 was added to the lower chamber of the Transwell cluster plates. After 6 hrs, cells migrating into the lower chamber were counted using a hemacytometer.

### Myeloma xenograft study

JJN3 cells (30 million cells/injection) were inoculated *s.c.* into the right flanks of nude mice (5-6 weeks old, female, Shanghai Slac Laboratory Animal Co. Ltd., Shanghai). When tumors were palpable, mice were randomly divided into two groups (n=5). One group was orally administrated C96 (100 mg/kg body weight, or 1/10 oral LD_50_) dissolved in PBS containing 10% Tween 80 and DMSO daily for continued 16 days, another group was received the vehicle only. Tumor sizes were measured every other day with a caliper and calculated according to the formula (V=0.5236*a²b*, where *a* is the smallest superficial diameter and *b* is the largest superficial diameter) [6, 36]. Body weight of each mouse was also monitored. At the end of the experiment, mice were sacrificed in a CO_2_ chamber containing dry ice. Tumor tissues were then excised, weighed, and snap-frozen in liquid nitrogen for further studies. To examine protein expression, tumor tissues were minced and ultrasonicated in the lysis buffer to extract proteins, which were further applied for immunoblotting analyses using specific antibodies against AKT, p-AKT, p-mTOR, mTOR, and GAPDH. This xenograft study was carried out in accordance with The Code of Ethics of the World Medical Association (Declaration of Helsinki) for experiments in animals and it was approved by the Animal Ethics and Welfare Review Board of Soochow University.

## SUPPLEMENTARY FIGURES AND TABLE



## Abbreviations

4E-BP1: eIF4E-binding protein 1
DMSO: dimethyl sulfoxide
FITC: fluorescein isothiocyanate
GAPDH: glyceraldehyde 3-phosphate dehydrogenase
IGF-1: insulin-like growth factor-1
IGF-1R: insulin-like growth factor-1 receptor
IMDM: Iscove's modified Dulbecco's medium
MM: multiple myeloma
mTOR: mammalian target of Rapamycin
MTT: 3-(4,5-dimethylthiazol-2-yl)-2,5-diphenyltetra-sodium bromide
PARP: poly(ADP-ribose) polymerase
PDB: protein data bank
PI: propidium iodide
PI3K: phosphatidylinositol 3-kinase
PTEN: phosphatase and tensin homolog
SDS: sodium dodecyl sulfate
TBS: Tris-buffered saline.
