# Supplementary material for: A virtual screen identified C96 as a novel inhibitor of phosphatidylinositol 3-kinase that displays potent preclinical activity against multiple myeloma in vitro and in vivo
Source: Oncotarget. 2014 May 22;5(11):3836–48. doi: 10.18632/oncotarget.1657 (PMC4116524; doi:10.18632/oncotarget.1657)
Supplement: Supplementary file 1 [file oncotarget-05-3836-s001.pdf]

SUPPLEMENTARY FIGURES AND TABLE

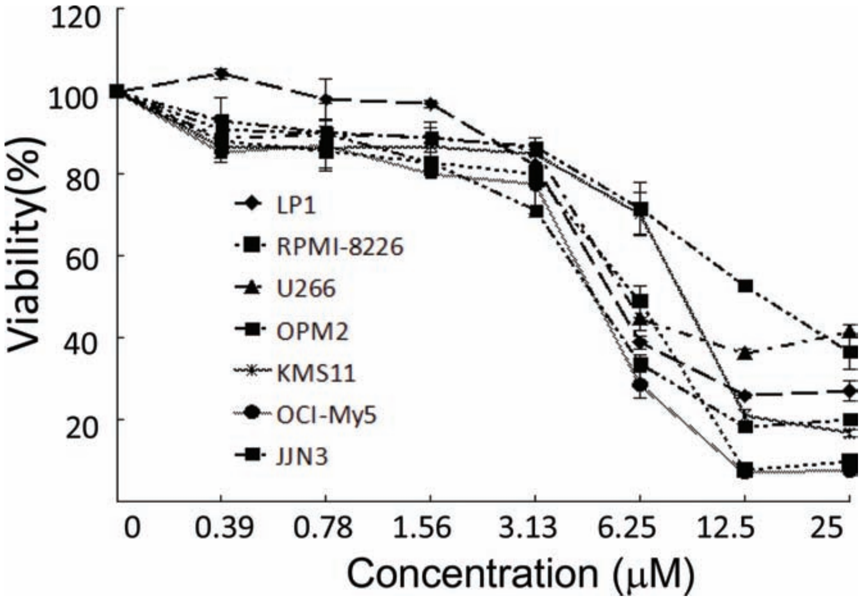

Supplementary Figure S1: C96 inhibits multiple myeloma cell proliferation. MM cell lines were treated with C96 with indicated concentrations for 72 hrs, followed by MTT assay.

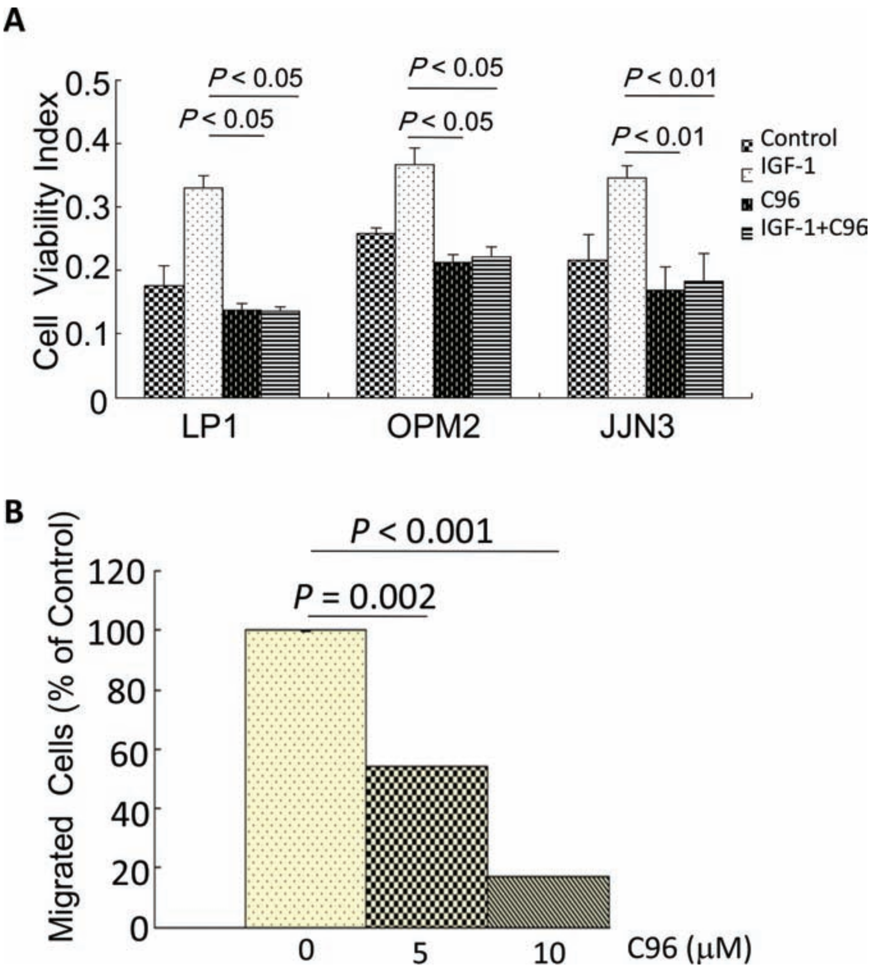

Supplementary Figure S2: C96 inhibits MM cell proliferation and migration in the presence of IGF-1. **(A)** MM cell lines LP1, OPM2, and JJN3 were maintained in IMDM media containing 0.5% FBS for 12 hrs, followed by C96 treatment alone or in combination with 100 ng/ml of IGF-1. Cells were then incubated for another 24 hr, cell number was analyzed using MTT staining. **(B)** LP1 cells were serum-starved overnight and then re-suspended in 70 μL of IMDM medium/0.5% fetal calf serum with or without C96. Cells were loaded onto the upper chamber of a transwell, and the lower chamber was filled with IMDM containing agonist IGF-1.After 6 hrs, cells migrating into the lower chamber were counted using a hemacytometer.

Supplemental Table 1: C96 fails to inhibit AKT and mTOR catalytic activity

Conc (M) of C96	AKT1	AKT2	AKT3	mTOR
3.00E-04	57.61%	86.10%	79.18%	102.57%
1.00E-04	89.13%	910.98%	98.26%	101.78%
3.33E-05	100.86%	100.38%	108.59%	100.02%
1.11E-05	104.72%	102.16%	100.98%	102.93%
3.70E-06	110.72%	102.25%	117.76%	101.74%
1.23E-06	101.71%	97.18%	100.31%	99.81%
4.12E-07	99.76%	100.48%	106.62%	99.37%
1.37E-07	102.27%	97.27%	100.29%	100.28%
4.57E-08	103.80%	100.26%	102.54%	99.19%
1.52E-08	94.32%	95.67%	109.49%	96.95%
DMSO	96.79%	100.91%	100.12%	98.54%
